# Exploring the diagnostic potential of plasma circ-CCDC66 in colorectal cancer

**DOI:** 10.1038/s41598-025-95685-5

**Published:** 2025-04-03

**Authors:** Zhuoting Han, Lok Ting Chu, Xiaocong Lin, Tao Zeng

**Affiliations:** 1 Laboratory Medicine Center, Affiliated Hospital of Guangdong Medical University, Zhanjiang, Guangdong P. R. China; 2 Laboratory Medicine Department, The Second Affiliated Hospital of Guangdong Medical University, Zhanjiang, Guangdong P. R. China; 3 Institute of Biochemistry and Molecular Biology, Guangdong Medical University, Zhanjiang, Guangdong P. R. China

**Keywords:** Colorectal cancer, Diagnostic biomarkers, Plasma, *Circ-CCDC66*, Intestinal polyp, Cancer, Genetics

## Abstract

Owing to the subtle symptoms and low sensitivity of colorectal cancer (CRC), such as rectal bleeding, anemia, and abdominal pain, CRC generally occurs in late stages. This leads to delayed diagnosis and reduced treatment efficiency. Therefore, novel and effective CRC markers are needed. In this study, we conducted circRNA expression profiling on GSE datasets and identified *circ-CCDC66* as a potential marker. Through qPCR analysis of plasma samples from 50 CRC patients, 50 with colorectal polyps, and 50 healthy individuals, *circ-CCDC66* emerged as a promising biomarker for CRC detection capable of identifying polyps among healthy individuals. Furthermore, plasma *circ-CCDC66* levels exhibited superior diagnostic accuracy, with an AUC of 0.920, compared to traditional markers, such as serum CEA and CA19-9. Remarkably, the combined detection of circ-CCDC66, CEA, and CA19-9 further increased the AUC for distinguishing the colorectal polyps’ group from the healthy control group to 0.991, with sensitivity and specificity increasing to 98% and 96%, respectively. Additionally, a *circ-CCDC66*-miRNA-mRNA regulatory network was established to provide insight into how circ-CCDC66 influences CRC pathways. This pioneering study highlights circ-CCDC66’s clinical relevance as a blood-based diagnostic marker for CRC and offers valuable insights into future developments in the diagnosis and treatment of the disease.

**Supplementary Information:**

The online version contains supplementary material available at 10.1038/s41598-025-95685-5.

## Introduction

Colorectal cancer (CRC), a common malignant tumor of the digestive tract with a high incidence rate and mortality^1–3^, ranks third in terms of incidence rate and second in terms of mortality in China, according to the 2020 Cancer Statistics Report^2^. Obvious symptoms such as rectal bleeding, anemia, and abdominal pain generally occur in the late stages of CRC, leading to delayed diagnosis and reduced treatment efficiency. This increases the probability of nerve invasion and lymph node metastasis^4^. Thus, early diagnosis is crucial as it is the best approach to prevent and avoid adverse prognoses^5^.

On the other hand, polyps are associated with precancerous lesions^6^. Colorectal adenomas are the most common precancerous colon lesions. Most CRCs originate from precancerous polyps^7^. If there is familial adenomatous polyposis, the risk of developing CRC can often become higher^8,9^. The progression from adenoma to adenocarcinoma takes approximately 10–15 years. Having sufficient time and opportunity to implement intervention measures could prevent the progression of intestinal polyps^10^. To solve these problems, there is a need to develop a method that can distinguish patients with intestinal polyps from healthy individuals and identify the presence of CRC at an early stage. Unfortunately, the clinical symptoms of polyps and early CRC are atypical, and their detection rates are low^11^. Currently, there are no clinically available screening indicators with high diagnostic efficiency at present^12,13^.

Currently, colonoscopy is the standard clinical CRC diagnosis method^14–16^. Colonoscopy is invasive, and, because colonoscopy cannot be retested in a short period and the quality of diagnostic results may be affected by the operator’s skill, it cannot be widely used as a screening method for early diagnosis^17^. Currently, carbohydrate antigen 199 (CA19-9) and carcinoembryonic antigen (CEA)^18,19^ are considered serological indicators for assisting in the diagnosis of CRC, but they cannot distinguish between colon polyps and healthy controls^20^. Therefore, there is great significance in finding new and efficient early diagnostic markers to improve the early detection rate of CRC, leading to a reduction in its incidence and mortality rates^21^.

Circular RNA (circRNAs) are an emerging class of endogenous non-coding RNA molecules^22,23^. CircRNAs regulate gene expression at the transcriptional, post-transcriptional, and translational levels and have various biological functions^24–26^. In recent years, many circRNAs have been reported to be related to CRC and are widely involved in cancer processes, such as cell proliferation, differentiation, apoptosis, invasion, and migration^27–29^. They also play important roles in the occurrence, development, and prognosis of CRC^29–31^. The gene *circ-CCDC66*, also known as hsa_circ_0001313, is an exon circRNA molecule formed by the reverse splicing of exons 8–10 of its parent gene, the CCDC66 transcript^28^. *circ-CCDC66* is upregulated in various tumor tissues and cells, such as cervical, papillary thyroid, and gastric cancers, and plays an important role in their pathogenesis^32–34^. In cervical cancer, *circ-CCDC66*, a molecular sponge of miR-452-5p, can upregulate REXO1 expression by blocking the action of miR-452-5p to promote cell proliferation, migration, invasion, and accelerate cervical cancer progression^32^. In papillary thyroid carcinoma, *circ-CCDC66* upregulates LARP1 expression by binding to miR-129-5p, thereby enhancing cancer cell proliferation, migration, invasion, and transplanted tumor growth^33^. In gastric cancer, Circ-CCDC66 can accelerate the proliferation and invasion of gastric cancer cells by binding to miR-1238-3p. These reports confirm that *circ-CCDC66* has the potential to become a biomarker for cancer^34^.

To date, there have been no reports on the clinical application of *circ-CCDC66* in patients with CRC, and it has not been confirmed whether *circ-CCDC66* can be used as a serological diagnostic marker. In this study, we evaluated the potential of *circ-CCDC66* as a biomarker from two aspects: in vitro detection analysis using blood plasma samples and building *circ-CCDC66*-miRNA-mRNA regulatory network using bioinformatic techniques. First, we comprehensively applied qPCR, chemiluminescence detection, and ROC curve analyses to evaluate the clinical value of plasma *circ-CCDC66* levels with respect to CRC diagnosis and its potential value in combined diagnosis with CEA and CA19-9. We found that the diagnostic efficacy (AUC) of plasma *circ-CCDC66* combined with CEA and CA19-9 in intestinal polyps achieved 0.97 (sensitivity of 92%; specificity of 94%), which was the highest AUC among other reported *circ-CCDC66*. Compared to the healthy control group, plasma *circ-CCDC66* levels were significantly upregulated in patients with CRC and CEA- and CA19-9-negative results. This indicates that *circ-CCDC66* could serve as a biomarker for detecting CRC and may even identify the presence of colorectal polyps in healthy individuals. Simultaneously, we applied bioinformatics techniques to analyze the expression levels of *circ-CCDC66* in CRC tissue samples and obtained target miRNAs and mRNA related to *circ-CCDC66* through target gene prediction. We successfully constructed a *circ-CCDC66*-miRNA-mRNA regulatory network and conducted correlation analysis. KEGG pathway enrichment analysis and GO gene functional enrichment analysis further revealed that *circ-CCDC66* may be involved in the biological processes and signaling pathways of CRC, providing new potential diagnostic biomarkers and therapeutic target genes for the disease. These results confirmed that *circ-CCDC66* has the potential to become a peripheral blood diagnostic marker for CRC.

## Results

### Identification of circ-CCDC66 through database acquisition and processing

**Fig. 1:**
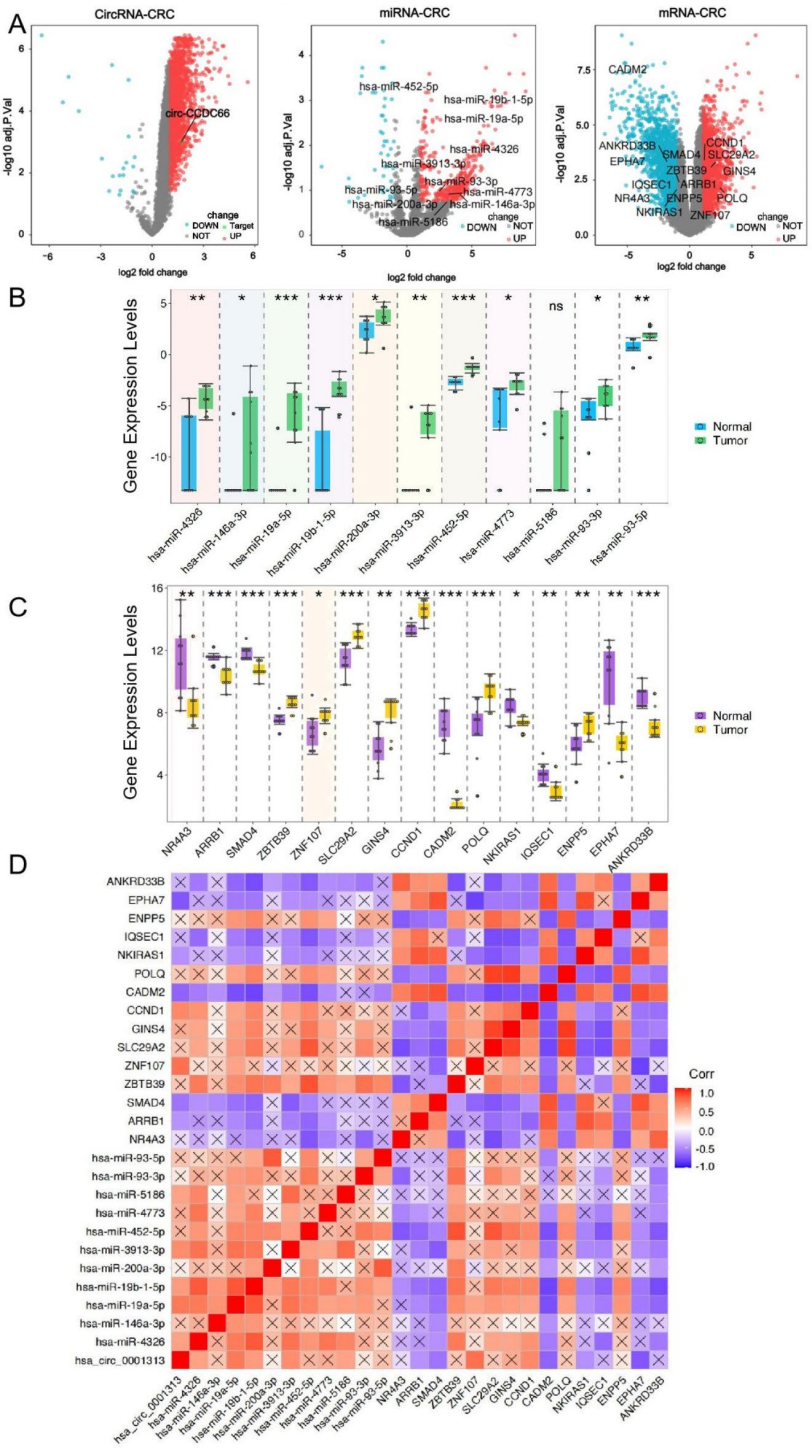
Expression profiles related to colorectal cancer from GSE datasets: (**A**) Volcano plot of circular ribonucleic acid (left), miRNA (middle), mRNA (right) expression levels. (**B**) Target miRNA of *circ-CCDC66* in colorectal cancer obtained from the GSE126093 dataset. (**C**) *circ-CCDC66* downstream target mRNA in colorectal cancer generated from the GSE 126,092 dataset. (**D**) Correlation analysis of *circ-CCDC66* (has_circ_0001313) and nodes of its related miRNA and mRNA network by Pearson correlation analysis. Red represents a positive correlation, and purple represents a negative correlation.

Based on the Gene Expression Omnibus (GEO) database, we found that *circ-CCDC66* is a circRNA with increased expression in CRC (Fig. 1A, left). In addition, from the volcano plot generated based on the miRNA and mRNA expression profiles in the GSE126093 and GSE126092 datasets, we found that 441 miRNAs (Fig. 1A, middle) and 3276 mRNAs (Fig. 1A, right) were dysregulated in CRC tissues. Intersection analysis was performed between differentially expressed miRNAs related to CRC and the CSCD2.0 database for *circ-CCDC66*, whereas dysregulated mRNAs were paired with the miRWalk database. We identified 11 downstream target miRNAs closely related to *circ-CCDC66* (hsa-miR-4326, hsa-miR-146a-3p, hsa-miR-19a-5p, hsa-miR-19b-1-5p, hsa-miR-200a-3p, hsa-miR-3913-3p, hsa-miR-452-5p, hsa-miR-4773, hsa-miR-93-3p, hsa-miR-93-5p, and hsa-miR-5186) and 15 target mRNAs closely related to *circ-CCDC66*. Among them, eight mRNAs were downregulated (NR4A3, ARRB1, SMAD4, CADM2, NKIRAS1, IQSEC1, EPHA7, and ANKRD33B) and seven were upregulated (ZBTB39, ZNF107, SLC29A2, GINS4, CCND1, POLQ, and ENPP5). To visually display these targets, we plotted a box graph based on the differential expression levels (shown in Fig. 1B for miRNAs and Fig. 1C for mRNAs).

To investigate the correlation between *circ-CCDC66* and the predicted miRNA and mRNA nodes, we used Pearson’s correlation analysis to find the relationship and build a *circ-CCDC66*-miRNA-mRNA network. From Fig. 1D (detailed shown in Fig. S1), the results showed that *circ-CCDC66* was significantly positively correlated with hsa-miR-4326, hsa-miR-19b-1-5p, hsa-miR-19a-5p, hsa-miR-3913-3p, hsa-miR-5186, CCND1, and ZNF107; there is a significant negative correlation with SMAD4, ARRB1, CADM2, NKIRAS1, and EPHA7. This analysis made the same prediction as the correlated miRNAs and mRNAs listed in the intersection analysis above. A previous study showed that these differentially expressed miRNAs and mRNAs correlated with the development of CRC. This indicates that *circ-CCDC66* may participate in the occurrence and development of CRC by regulating these targets, which provides a new perspective to understand the potential biological function of *circ-CCDC66* in the disease and to reveal the complex regulatory relationships between *circ-CCDC66* and miRNA and mRNA molecules.

### Expression and diagnostic value of circ-CCDC66 in datasets and real samples

**Fig. 2:**
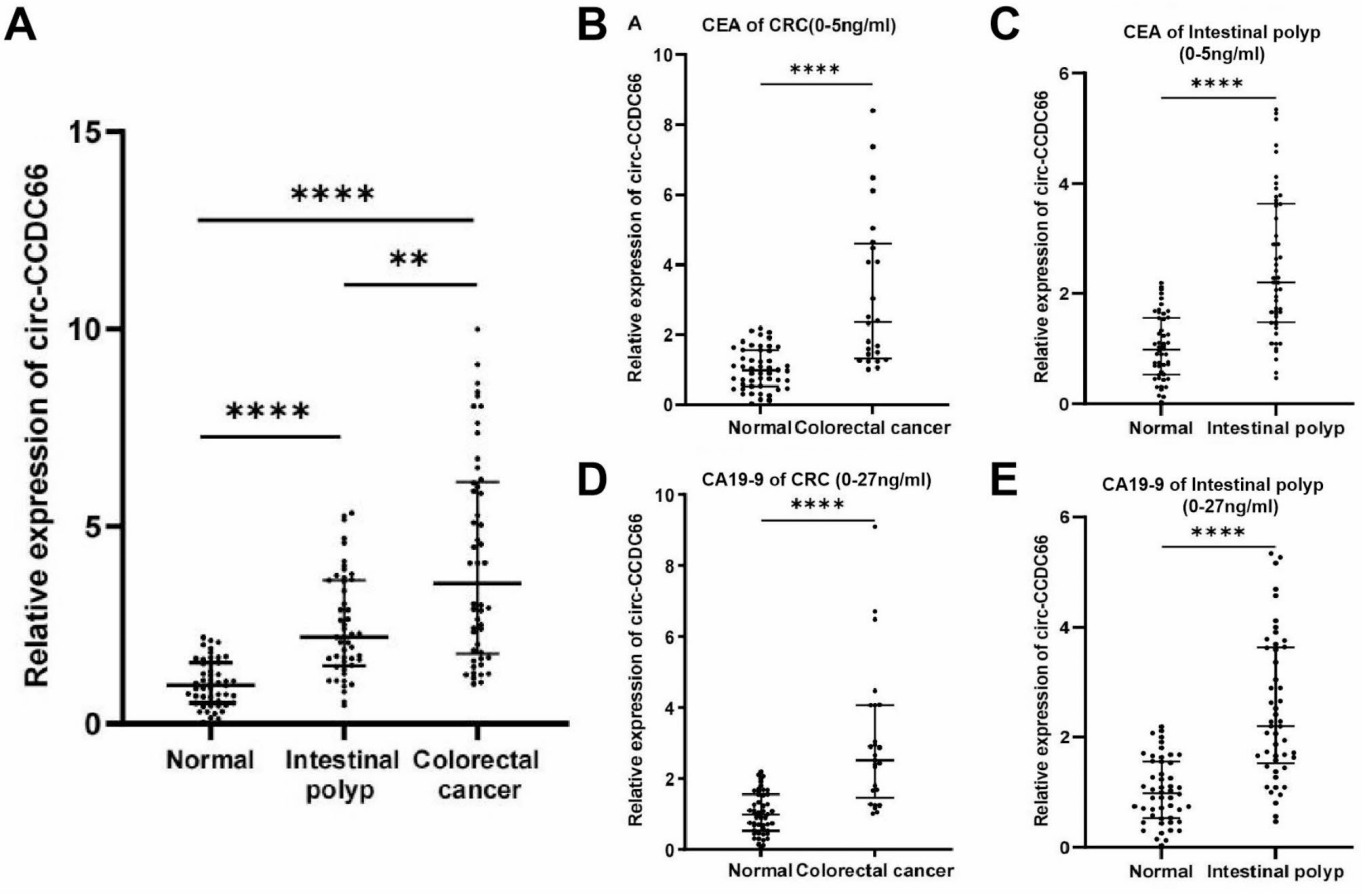
*Circ-CCDC66* expression levels in the plasma of different groups of patients: (**A**) Expression of *circ-CCDC66* in plasma of the normal healthy, intestinal polyp, and CRC groups. (**B**) *Circ-CCDC66* expression levels in patients with normal CEA within the CRC group. (**C**) Expression levels of *circ-CCDC66* in patients with normal CEA in the intestinal polyp group. (**D**) Expression levels of *circ-CCDC66* in patients with normal CA19-9 within the CRC group. (**E**) Expression levels of *circ-CCDC66* in patients with normal CA19-9 in the intestinal polyp group. Statistical significance is indicated by ns (*p* > 0.05), * (*p* < 0.05), ** (*p* < 0.01), *** (*p* < 0.001), and **** (*p* < 0.0001).

Next, we analyzed circ-CCDC66 tissue samples in the GSE126094 database. The results revealed that the expression of circ-CCDC66 was significantly upregulated in CRC tissue samples compared to adjacent tissues (Fig. S2). According to pathological diagnoses, intestinal polyps include adenomatous, proliferative, and inflammatory polyps. Adenomatous intestinal polyps had the strongest correlation with CRC, and are considered precancerous lesions. Thus, we conducted expression analysis of clinical plasma samples using RT-qPCR, including the healthy normal, intestinal polyp, and CRC groups. Compared to the healthy control group, there was an increase in the expression of *circ-CCDC66* in the plasma samples of the intestinal polyp group, and the expression was higher in CRC (Fig. 2A). In addition to the classification based on clinical information from the hospital results, we conducted a chemiluminescence assay for the expression of CA19-9 and CEA, as shown in Fig. S3, which are the clinical markers of CRC, to confirm whether these samples were related to CRC. The CRC samples showed high CEA and CA19-9 expression levels. However, we noticed that it was difficult to distinguish patients with intestinal polyps from normal individuals based on CA19-9 and CEA levels. Furthermore, patients classified as having intestinal polyps or CRC based on the hospital clinical information may exhibit normal CEA and CA19-9 expression levels. In detail, 50% of patients with CRC (25 out of 50) had normal CEA and CA19-9 levels, and almost all patients with intestinal polyps had normal CEA or CA19-9 levels (46 out of 49 and 50, respectively). This may reduce the accuracy of diagnosis and the chances of early detection and treatment. However, we revealed that these patients with intestinal polyps or CRC and normal CEA or CA19-9 levels had higher *circ-CCDC66* expression (based on RT-qPCR results shown in Fig. 2B-E). This reveals that *circ-CCDC66* can be used for more accurate discrimination between patients with intestinal polyps or CRC and normal CEA/CA19-9 levels from healthy controls.

**Fig. 3:**
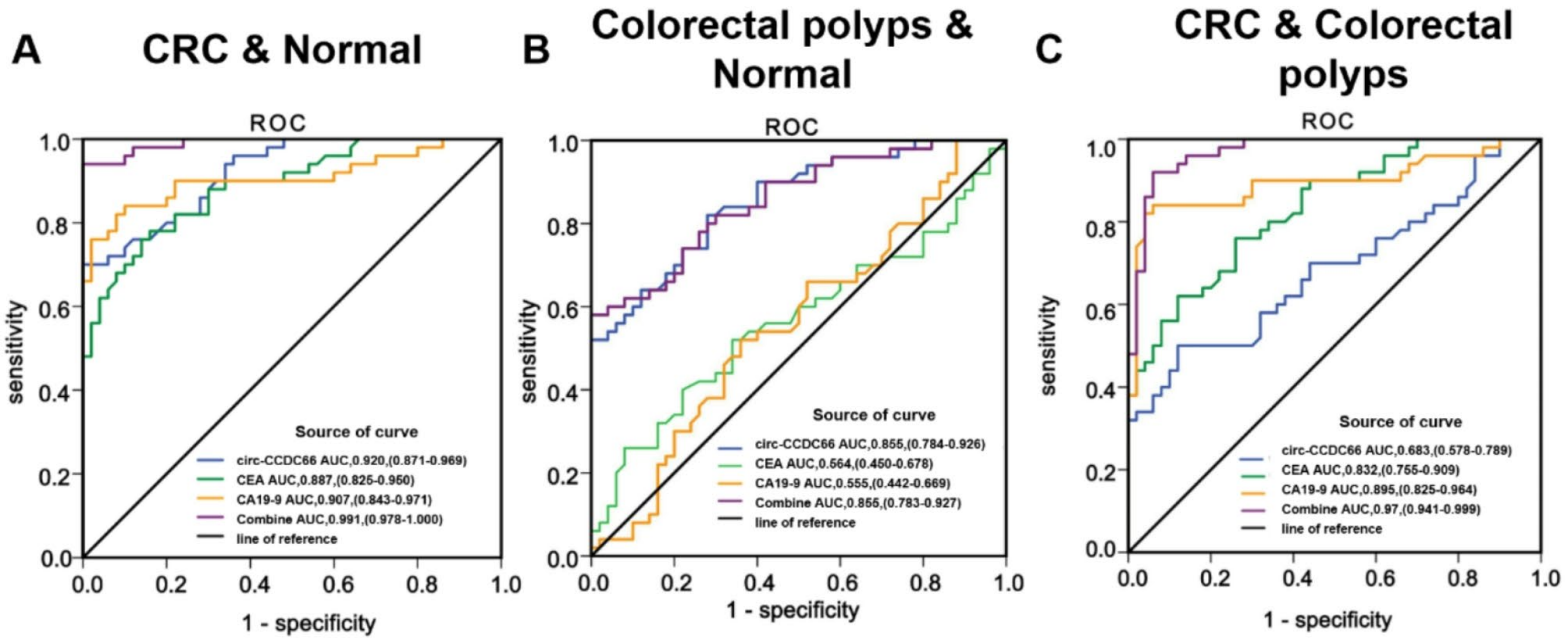
ROC curves of single and combined detection of *circ-CCDC66*, CA19-9, and CEA in different diagnoses: (**A**) For the diagnosis of colorectal cancer among the healthy group. (**B**) For the diagnosis of colorectal polyps among the healthy group. (**C**) For the diagnosis of colorectal cancer among colorectal polyps.

Next, we studied the static diagnostic efficacy based on the ROC curve analysis shown in Fig. 3 and Table S1. The ROC curve drawn according to the predicted probability and actual value is shown in Fig. 3. The highest cut value of the Youden index (sensitivity + specificity − 1) was calculated, and the corresponding sensitivity and specificity were obtained from the best cut-off point. The AUC of plasma *circ-CCDC66* levels distinguishing the CRC group from the healthy control group was 0.920, with a sensitivity of 94% and a specificity of 72%. The diagnostic efficacy was better than the current detection markers (serum CEA 88.7%, CA19-9 90.7%). Detailed information is provided in the Supporting Information (Table S5). This indicated that *circCCDC66* had a significant advantage in the prediction of CRC. Remarkably, the combined detection of *circ-CCDC66*, CEA, and CA19-9 further increased the AUC for distinguishing the colorectal polyp group from the healthy control group to 0.991, with sensitivity and specificity increased to 98% and 96%, respectively. This suggests that the combined detection of *circ-CCDC66*, CEA, and CA19-9 effectively improved the diagnostic efficacy of CRC. Meanwhile, the AUC for distinguishing intestinal polyps from the group with colorectal polyps and the healthy control group was 0.855 (sensitivity, 82%; specificity, 72%). In contrast, the combined diagnosis did not improve (AUC = 0.855; sensitivity, 74%, specificity, 78%), compared with measuring *circ-CCDC66* alone. Importantly, the AUC for distinguishing CRC from colorectal polyps was 0.683 (sensitivity, 50%; specificity, 88%), whereas the combination of three markers increased the AUC to 0.97 (sensitivity, 92%; specificity, 94%).

Additionally, we reviewed the performance of other circRNAs and compared them with *circ-CCDC66*. As shown in Table 1, *circ-CCDC66* achieved the highest score in both AUC and sensitivity compared to others, and its performance improved to > 95% in all aspects (AUC, sensitivity, and specificity) with the combination treatment (*circ-CCDC66* + CEA + CA19-9). Furthermore, we conducted a comparison between circ-CCDC66 and other CRC molecular diagnostic markers, including mRNA, miRNA, and lncRNA ( Table S6), and found that circ-CCDC66 could provide a promising AUC. This indicates that the detection of *circ-CCDC66* effectively enhanced the diagnostic efficiency for colorectal diseases (including intestinal polyps and CRC). Combined detection with other markers, such as CEA and CA19-9, helped reduce missed diagnoses and misdiagnoses, providing a more reliable basis for clinical diagnosis and treatment.

**Table 1:** Diagnostic efficacy comparison among different circrnas in CRC.

CircRNA	AUC	Sensitivity	Specificity	Ref
circ_0004771	0.86	81.4%	81.4%	^30^
circZNF609	0.767	65.2%	65.2%	^31^
circGAPVD1	0.7662	75.64%	71.79%	^29^
hsa_circ_0006282	0.75	71.2%	80.1%	^35^
CEA	0.59	48.3%	77.2%	
CA19-9	0.531	29.1%	77.8%	
hsa_circ_0006282 + CEA	0.80	79.3%	77.8%	
hsa_circ_0006282 + CA19-9	0.791	63.8%	88.1%	
hsa_circ_0006282 + CEA + CA19-9	0.79	78.8%	76.9%	
CircPanel	0.874	67.65%	90.00%	^36^
CEA	0.724	63.73%	80.00%	
CircPanel + CEA	0.903	82.35%	83.75%	
hsa_circ_0124554	0.742	93.8%	46.9%	^37^
hsa_circ_0124554 + CEA	0.886	81.2%	81.2%	
hsa_circ_0124554 + CA19-9	0.817	84.4%	71.9%	
hsa_circ_0124554 + CEA + CA19-9	0.899	84.4%	84.4%	
Circ-SMARCA5	0.611	90.0%	70.0%	^38^
Circ-NOL10	0.803	70.0%	95.0%	
Circ-LDLRAD3	0.768	64.0%	95.0%	
Circ-RHOT1	0.836	70.0%	95.0%	
CEA	0.878	76.0%	95.0%	
hsa_circ_0026416	0.767	/	/	^39^
CEA	0.670	/	/	
CA19-9	0.592	/	/	
CA72-4	0.575	/	/	
circ-CCDC66	0.920	94%	72%	Our work
CEA	0.887	88%	70%	
CA19-9	0.907	90%	78%	
*circ-CCDC66* + CEA + CA19-9	0.991	98%	96%	

We also used the chi-squared test to analyze the relationship between plasma *circ-CCDC66* levels and various clinical and pathological characteristics of patients with CRC. The results showed that the expression level of plasma *circ-CCDC66* was not significantly correlated with the age and gender of patients but was significantly positively correlated with lymph node metastasis, nerve invasion, tumor size, and TNM staging (Table S7).

**Fig. 4:**
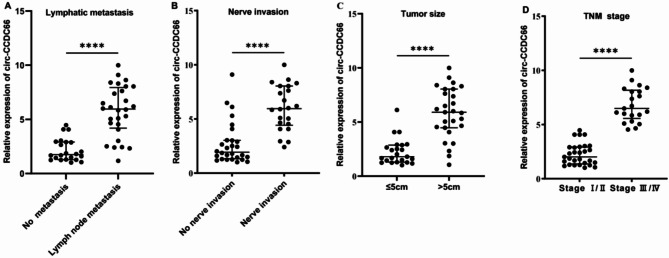
Expression of plasma *circ-CCDC66* in different pathological features related to the progression and malignancy of patients with colorectal cancer: (**A**) lymphatic metastasis; (**B**) nerve invasion; (**C**) tumor size; and (**D**) TNM stage. Statistical significance was symbolized by ns (*p* > 0.05), * (*p* < 0.05), ** (*p* < 0.01), *** (*p* < 0.001), and **** (*p* < 0.0001).

Based on the expression analysis, we categorized patients with CRC based on whether lymph node metastasis occurred (22 cases without and 28 cases with metastasis), nerve invasion occurred (28 cases without and 22 cases with nerve invasion), tumor size (26 cases < 5 cm, 24 cases > 5 cm), and TNM staging (28 cases were stage I-II, 22 cases were stage III-IV). The RT-qPCR results showed that the plasma *circ-CCDC66* levels in patients with lymph node metastasis were higher than those without (*p* < 0.001, see Fig. 4A); patients with nerve invasion had higher levels than those without (*p* < 0.001, see Fig. 4B); patients with a tumor diameter > 5 centimeters had higher levels than those with a tumor diameter < 5 centimeters (*p* < 0.001, see Fig. 4C), and the plasma *circ-CCDC66* levels in patients with stage III-IV were also higher than those in patients with stage I-II (*p* < 0.001, see Fig. 4D). The results showed that the expression level of plasma *circ-CCDC66* was significantly and positively correlated with lymph node metastasis, nerve invasion, tumor size, and TNM staging. This suggests that *circ-CCDC66* may play a role in the development, invasion, and metastasis of CRC.

### Construction of *circ-CCDC66*-miRNA-mRNA regulatory networks and their pathway enrichment analysis

**Fig. 5:**
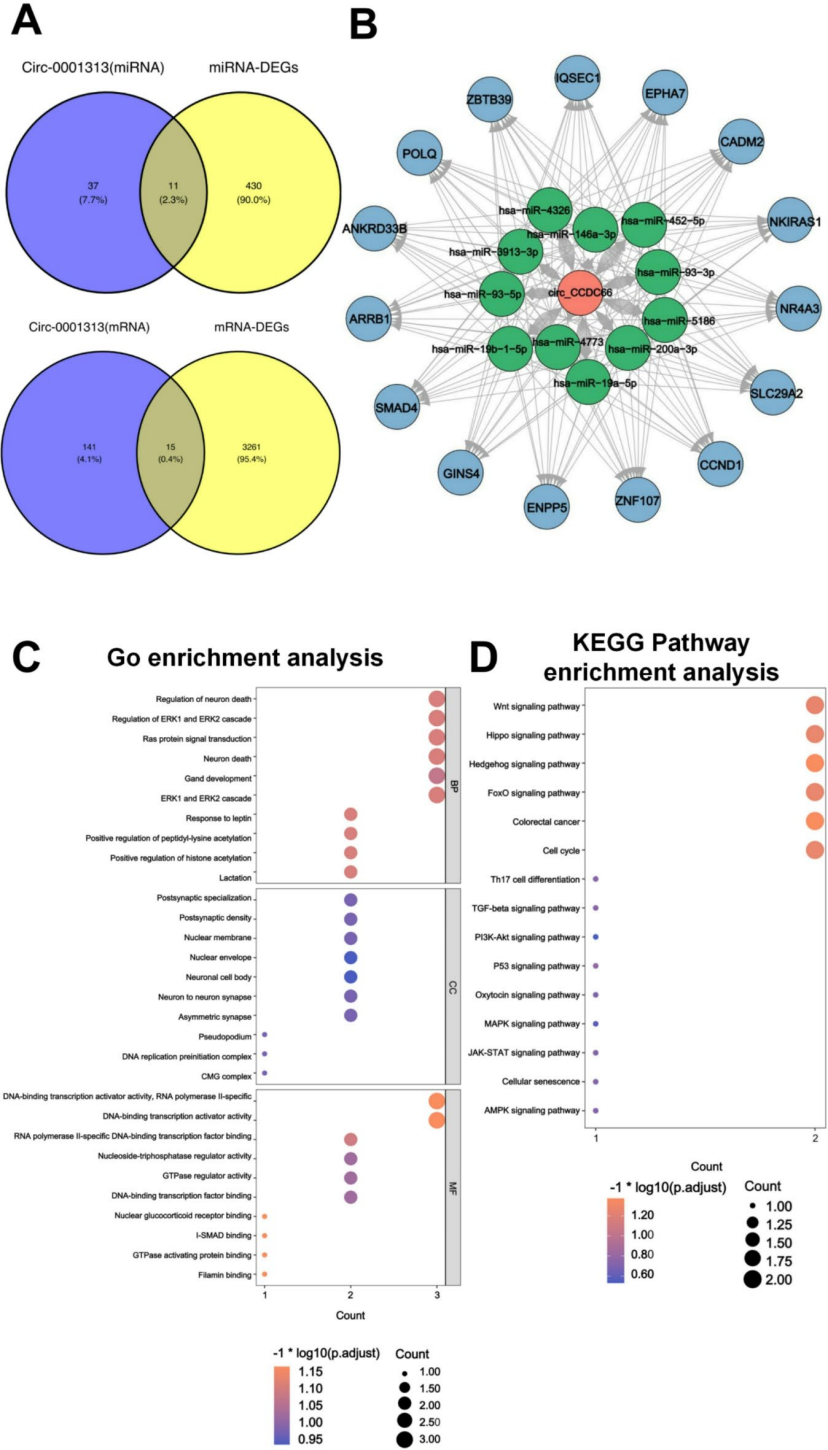
Construction of *circ-CCDC66*-miRNA-mRNA regulatory network: (**A**) Venn diagram predicting *circ-CCDC66* and its downstream closely related miRNA (top) and mRNAs (bottom). (**B**) Prediction of *circ-CCDC66*-miRNA-mRNA regulatory network diagram. (**C**) GO analysis of 15 target genes regulated by *circ-CCDC66*. (**D**) KEGG analysis of 15 target genes regulated by *circ-CCDC66* (**C**,**D**) Y-axis represents the name of the enrichment item, and the X-axis represents the ratio of the proportion of differentially expressed genes to the proportion of all genes to the enrichment item.

Based on the above analysis, we conclude that *circ-CCDC66* is a potential marker for the diagnosis of CRC. To provide more information for the development of *circ-CCDC66* applications, we used the ggvenn function in R language to predict the intersection between 48 downstream target miRNAs (left circle, Fig. 5A, top) and 441 DE miRNAs (miRNAs with differential expression) (right circle, Fig. 5A, top) related to CRC. The results showed that 11 miRNAs may be regulated by *circ-CCDC66* (2.3%). Furthermore, upon crossing with MiRWalk, 156 downstream target mRNAs were predicted to be closely related to 11 miRNAs. By intersecting these 156 mRNAs with 3276 differentially expressed mRNAs from CRC, 15 targeted mRNAs were predicted to be regulated by *circ-CCDC66* (0.4%) as shown in Fig. 5A, bottom. To gain a better understanding of the relationship between *circ-CCDC66* and its correlated genes, we constructed a ceRNA network using the Cytoscape software. A *circ-CCDC66*-miRNA-mRNA network, including *circ-CCDC66* and its regulated 11 target miRNAs and 15 target mRNAs, was constructed (Fig. 5B). In detail, *circ-CCDC66* regulated 11 miRNAs (green: hsa-miR-4326, hsamiR-146a-3p, hsa-miR-19a-5p, hsa-miR-19b-1-5p, hsa-miR-200a-3p, hsa-miR-3913-3p, hsa-miR-452-5p, hsa-miR-4773, hsa-miR-93-3p, hsa-miR-93-5p, and hsa-miR-5186) and 15 mRNAs (blue: NR4A3, ARRB1, SMAD4, CADM2, NKIRAS1, IQSEC1, EPHA7, ANKRD33B, ZBTB39, ZNF107, SLC29A2, GINS4, CCND1, POLQ, and ENPP5) in CRC. Hsa-miR-146a-3p [30], hsa-miR-200a-3p, hsa-miR-452-5p, and hsa-miR-93-5p play roles in the occurrence of various cancers. Within the nodes of the *circ-CCDC66*-miRNA-mRNA network, two miRNAs (18.18%) have been associated with intestinal diseases^40,41^; nine mRNAs (accounting for 60%, including NR4A3 ^42^, ARRB1 ^43^, SMAD4 ^44^, EPHA7 ^45^, CCND1 ^46,47^, and POLQ^48^) have been linked to the occurrence and development of CRC. As potential targets of miRNAs, the expression of mRNA can change owing to the direct or indirect dysregulation of miRNAs. Abnormal mRNA levels can alter cell function by affecting protein structure and controlling signaling pathways, leading to inhibition of cell growth and increased cell apoptosis. For example, in CRC, CCND1 expression significantly correlated with lymph nodes and distant metastases. It is closely related to a poor prognosis^49^. POLQ, expressed abnormally in many cancers, is a DNA repair enzyme linked to multiple types of cancer, and its overexpression is associated with a poor prognosis^50^. POLQ is associated with post-replication gap closure of mutations, which may drive the genomic evolution of cancer^51^. These studies indicate the potential significance of *circ-CCDC66* in the diagnosis and prognosis of CRC through its interaction with target miRNAs and mRNAs.

Next, we performed GO (Fig. 5C) and KEGG (Fig. 5D) enrichment analyses on mRNA nodes from our *circ-CCDC66*-miRNA-mRNA network. KEGG analysis showed that the network nodes were mainly enriched in 15 signaling pathways. Fifteen target genes were enriched in biological processes (BP), cell composition (CC), and molecular functions. Among these, 14 signaling pathways (93.33%) such as the Wnt, Hippo, and FoxO pathways, were associated with CRC progression. These signaling pathways are involved in the immune response, proliferation, metastasis, apoptosis, and other processes of CRC cells and play a crucial role in the occurrence and development of the disease. In contrast, GO analysis results showed that there were three of the 30 enriched items with differential genes. Among these, 13 hotspots related to biological processes (43.33%) have been studied and reported in CRC. These biological processes, such as the regulation and secretion of neurons, are related to the self-renewal of CRC stem cells and the acceleration of tumor formation^52^. Moreover, the cascade regulation of ERK1 and ERK2 is related to H3K9ac-mediated CRC development^53^. There is a correlation between CRC and biological processes such as RAS protein signal transduction^54^, glandular development^55^, and leptin-mediated responses^56^. Therefore, the GO and KEGG enrichment analysis results confirmed the reliability of the *circ-CCDC66*-miRNA-mRNA network, revealing a foundation for subsequent molecular mechanism studies.

## Discussion

Liquid biomarkers, such as circ-CCDC66, offer unique advantages, including ease of acquisition, non-invasiveness, high sensitivity, and specificity, providing new perspectives for disease diagnosis, targeted therapy, and prognosis. Although research on liquid biomarkers is still in its early stages, technological advancements and scientific innovations are expected to uncover new indicators.

The strength of this study lies in its novel exploration of circ-CCDC66’s medical value, particularly its potential as an early diagnostic biomarker for CRC. There are no previous reports on the expression of circ-CCDC66 in the plasma of patients with CRC and its clinical value. This study comprehensively used qPCR, chemiluminescence detection, and ROC curve analysis to evaluate the value of plasma circ-CCDC66 levels in the diagnosis of CRC. Additionally, the combined detection of circ-CCDC66, CEA, and CA 19 − 9 enhanced detection accuracy and reduced the chance of missed diagnoses and misdiagnoses in the early stages. Based on the literature review, we believe that the combined detection of new indicators and traditional diagnostic markers may improve performance and can be applied in clinical practice. Bioinformatics was used to analyze the expression of circ-CCDC66 in CRC tissue samples, predict target miRNAs and mRNAs, construct a circ-CCDC66-miRNA-mRNA regulatory network, and perform correlation and pathway enrichment analyses.

Furthermore, in CRC, the expression level of circ-CCDC66 was significantly different in the plasma of patients with lymph node metastasis, nerve invasion, tumor diameter greater than 5 cm, and advanced stage (stage III-IV). Therefore, the expression level of plasma circ-CCDC66 may have a potential auxiliary diagnostic role in CRC progression. These analyses revealed that circ-CCDC66 can act as a CRC biomarker for early diagnosis and prognosis.

However, research on the diagnostic value of plasma circ-CCDC66 in CRC is still in its early stages owing to the lack of detailed research on its downstream target genes and the derived network. Further research is needed to uncover the regulatory networks and explore additional mechanisms. Another limitation of this study is the insufficient sample size, which, despite showing statistically significant differences in circ-CCDC66 expression levels, could affect the generalizability of the findings. Moreover, the expression level of circ-CCDC66 is relatively quantitative and its copy number cannot be accurately measured, limiting its clinical laboratory application. Future studies will involve combining absolute quantitative methods and expanding the sample size to study circ-CCDC66 in a more comprehensive manner. The application field can also be broadened to explore its potential beyond early diagnosis, such as in combination with current treatments, such as repurposing drugs and vitamin D supplementation^57^.

## Materials and methods

### Blood sample collection

Peripheral blood samples from healthy people, patients with intestinal polyps, and patients with CRC were collected at The Second Affiliated Hospital of Guangdong Medical University and kept in anticoagulant ethylenediaminetetraacetic acid (EDTA)-coated tubes. The blood samples were centrifuged at 4000 ×g for 10 min to collect plasma and stored at −80ºC for subsequent experiments. To ensure the anonymity of the data, we labeled each sample with a unique code and correlated it with the experimental record. This ensured the confidentiality and privacy of the data. The study protocol was approved by the Ethics Committee of the Second Affiliated Hospital of Guangdong Medical University (document: PJKT2023-002), and all patients provided written informed consent. All experiments using these blood samples, including quantification by spectrophotometry and qPCR, were performed in accordance with relevant guidelines and regulations.

### Extraction and quantification of RNA from blood plasma

Total RNA was extracted from the plasma according to the manufacturer’s protocol for the magnetic bead-based nucleic acid extraction kit from Zybio (EXM6000 extractor). After extraction, 1.2% agarose gel running in TBE buffer was used to verify the integrity of RNA. The three bands in the gel represented intact RNA. RNA concentration was quantified using an ultra-trace spectrophotometer (ND_1000). A_260/A280_ values ranging from 1.8 to 2.0 indicate the good purity of RNA.

### Quantitative reverse transcription polymerase chain reaction (qRT-PCR)

Reverse transcription is conducted following the instruction of HiScript III RT SuperMix for qPCR. A negative control, excluding reverse transcriptase, was used to verify the presence of residual genomic DNA. Forward and reverse primers ( Table S1) were used to conduct the polymerase chain reaction. The test primers targeted the region of *circ-CCDC66*, and the glyceraldehyde-3-phosphate dehydrogenase (GAPDH) primers acted as the endogenous control. Detailed information is provided in the supplemental information (Tables S2, S3). This process was performed using a CXF96 fluorescence quantitative PCR instrument. Relative quantitative calculation was performed using the 2^−ΔΔct^ method to present the difference in gene expression levels between the experimental group and the control group.

### Bioinformatics data collection and analysis

We downloaded three datasets (GSE126094, GSE126093, and GSE126092) from the GEO database to perform bioinformatics analysis (website link shown in Table S4). The GSE126094 dataset contains circRNA expression profiles of 10 CRC tissues and their corresponding normal tissues (NAT). The GSE126093 dataset contains the miRNA expression profiles of 10 CRC tissues and their corresponding normal tissues (NAT). The GSE126092 dataset contains the lncRNA/mRNA expression profiles of 10 CRC tissues and their corresponding normal tissues (NAT). In this study, all the research objects were collected from open online databases.

To understand the differential expression (DE) among circRNAs, miRNAs, and mRNAs in CRC, as well as explore the downstream target miRNAs and mRNAs of *circ-CCDC66* in CRC, we used the Limma and ggplot function in R language (https://www.rproject.org/) and Bioconductor (http://www.bioconductor.org/) to identify DE circRNAs, DE miRNAs, and DE mRNAs. The GPL18058 platform was used to analyze the GSE126093 and GSE126092 datasets and ggplot boxplots were drawn to display their differential expression. LogFC ≥ | 1 | and *P* < 0.05 were used as cut-off values for selecting DE miRNAs and DE mRNAs. Finally, gene correlation analysis was performed on the *circ-CCDC66* miRNA-mRNA network using the downstream derivatives of *circ-CCDC66*.

We used the cancer-specific CSCD2.0 database (http://gb.whu.edu.cn/CSCD2/#) to predict the downstream miRNAs of *circ-CCDC66*. Using the R language function ggvenn, we determined the intersection between the predicted and DE miRNAs. After predicting the downstream targeted miRNAs related to *circ-CCDC66*, we predicted the corresponding targeted mRNA using the miRWalk database. The intersection between miRNA-predicted mRNAs and DE mRNAs was selected to build a circ-CCDC66-miRNA-mRNA interaction regulatory network closely related to the occurrence and development of CRC using Cytoscape version 3.8.0 (https://cytoscape.org/).

### Statistical analysis

Statistical analyses and comparisons were performed using (GraphPad Prism 8.0 and SPSS 23.0). Mann–Whitney’s non-parametric test was used for data with a non-normal distribution. When *p* < 0.05, the differences among the groups were considered significant. Statistical significance was indicated by ns (*p* > 0.05), * (*p* < 0.05), ** (*p* < 0.01), *** (*p* < 0.001), and **** (*p* < 0.0001).

## Conclusion

In this study, we hypothesized the biological value of *circ-CCDC66* in CRC based on an analysis of the GEO database. Through clinical plasma sample verification, we found that *circ-CCDC66* was more highly expressed in colorectal polyps and CRC than in healthy controls. Furthermore, the combined detection of plasma *circ-CCDC66*, serum CA19-9, and serum CEA levels improved the diagnostic efficacy for CRC, leading to higher AUC values, sensitivity, and specificity. Finally, we constructed a *circ-CCDC66-*miRNA-mRNA regulatory network to determine the potential interactions between *circ-CCDC66* and related miRNAs and target mRNAs. The results of the KEGG pathway and GO gene functional enrichment analyses further highlighted the involvement of *circ-CCDC66* in the biological processes and signaling pathways of CRC. Based on these results, we suggest that plasma *circ-CCDC66* can serve as a peripheral blood diagnostic marker for CRC.

## Electronic supplementary material

Below is the link to the electronic supplementary material.

Supplementary Material 1

## Data Availability

The experimental data of this work are available from the corresponding author with reasonable request.

## Abbreviations

AUC: Area under the curve
BP: Biological process
CA19-9: Carbohydrate antigen 19-9
CC: Cell composition
CEA: Carcinoembryonic antigen
CircRNA: Circular RNA
CRC: Colorectal cancer
Ct: Cycle threshold
DE: Differential expression
EDTA: Ethylenediaminetetraacetic acid
GAPDH: Glyceraldehyde-3-phosphate dehydrogenase
GEO: Gene expression omnibus
NAT: Normal tissues
QPCR: Quantitative real-time polymerase chain reaction
ROC: Receiver operating characteristic
