# Supplementary material for: Exploring the diagnostic potential of plasma circ-CCDC66 in colorectal cancer
Source: Sci Rep. 2025 Apr 3;15:11463. doi: 10.1038/s41598-025-95685-5 (PMC11968963; doi:10.1038/s41598-025-95685-5)
Supplement: Supplementary file 1 — Supplementary Material 1 [file 41598_2025_95685_MOESM1_ESM.docx]

### Exploring the Diagnostic Potential of Plasma circ-CCDC66 in Colorectal Cancer

### Zhuoting Han^1,2^, Lok Ting Chu^1,3^, Xiaocong Lin^1,3^, Tao Zeng^1*^

^1^Laboratory Medicine Center, Affiliated Hospital of Guangdong Medical University, Zhanjiang, Guangdong, P. R. China.

^2^Laboratory Medicine Department, The Second Affiliated Hospital of Guangdong Medical University, Zhanjiang, Guangdong, P. R. China.

^3^Institute of Biochemistry and Molecular Biology, Guangdong Medical University, Zhanjiang, Guangdong, P. R. China.

Table S 1Gene primer sequences

| Gene |  | Sequence |
| --- | --- | --- |
| *Circ-CCDC66* | Forward (5’-3’) | CGAGACAGACGACGACAAAA |
| *Circ-CCDC66* | Reverse (5’-3’) | TTGACGGTCATCTTCTATTTGC |
| *GAPDH* | Forward (5’-3’) | GAAACTGTGGCGTGATGGC |
| *GAPDH* | Reverse (5’-3’) | CACCACTGACACGTTGGCAG |

Table S 2 RT-qPCR reaction system

| Reagent | Volume（μL） |
| --- | --- |
| S2 x ChamQ Universal SYBR qPCR Master Mix | 10.0 μL |
| Forward Primer（10 μM） | 0.4 μL |
| Reverse Primer（10 μM） | 0.4 μL |
| cDNA | 1.0 μL |
| RNase Free dH_2_O | Up to 20 μL |
| Total | 20.0 μL |

Table S 3 RT-qPCR reaction program setting

| Stage | Reaction | Cycle No. | Temperature | Time |
| --- | --- | --- | --- | --- |
| Stage 1 | Pre-denaturation | Reps: 1 | 95℃ | 30 sec |
| Stage 2 | Chain reaction cycle | Reps: 40 | 95℃  60℃ | 10 sec  30 sec |
| Stage 3 | Melting Curve | Reps: 1 | 95℃  60℃  95℃ | 15 sec  60 sec  15 sec |

Table S 4 databases links

|  | Website link |
| --- | --- |
| Gene Expression Omnibus | http://www.ncbi.nlm.nih.gov/geo/ |
| circRNA (GSE126094) | https://www.ncbi.nlm.nih.gov/geo/query/acc.cgi?acc=GSE126094 |
| microRNA  (GSE126093) | https://www.ncbi.nlm.nih.gov/geo/query/acc.cgi?acc=GSE126093 |
| mRNA  (GSE126092) | https://www.ncbi.nlm.nih.gov/geo/query/acc.cgi?acc=GSE126092 |


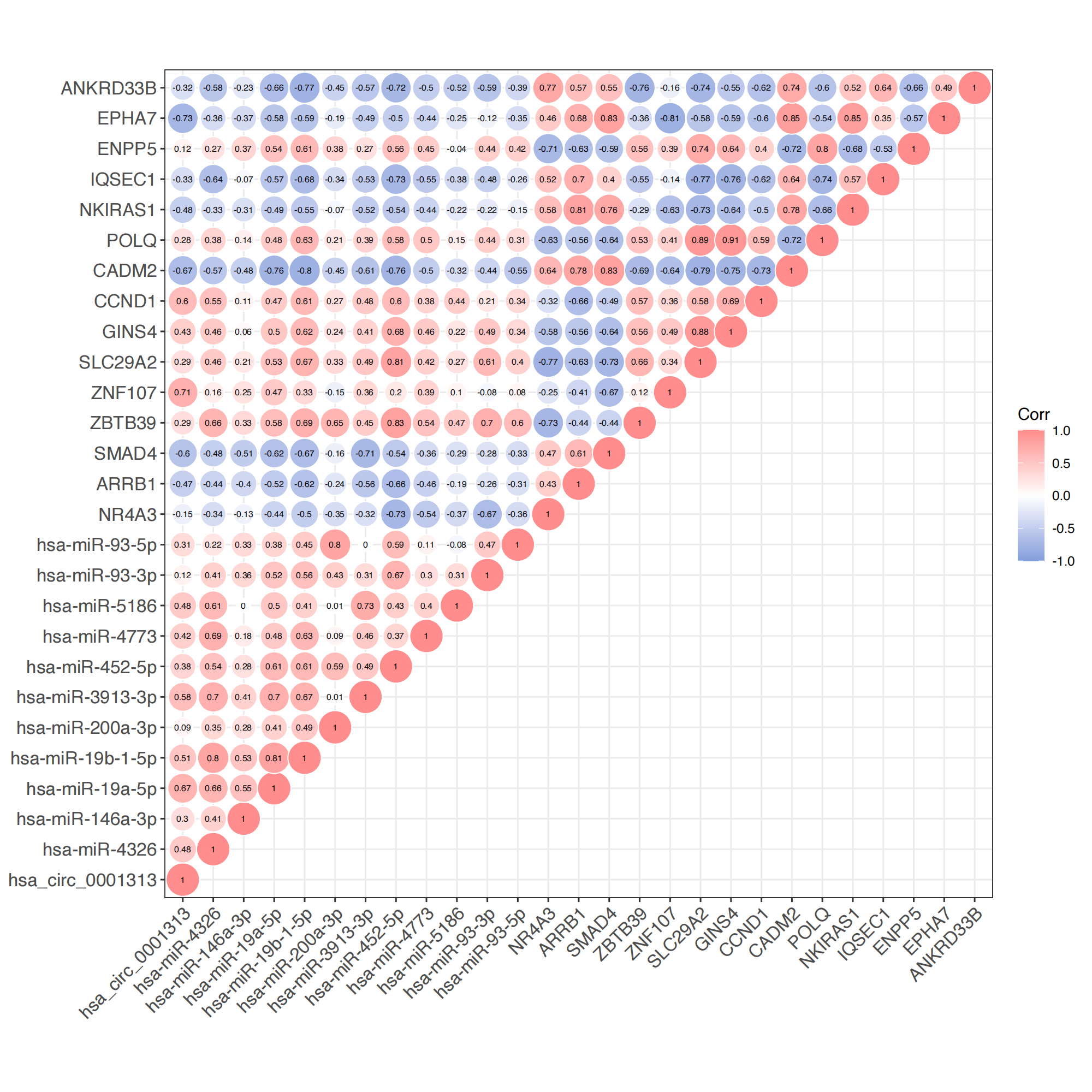

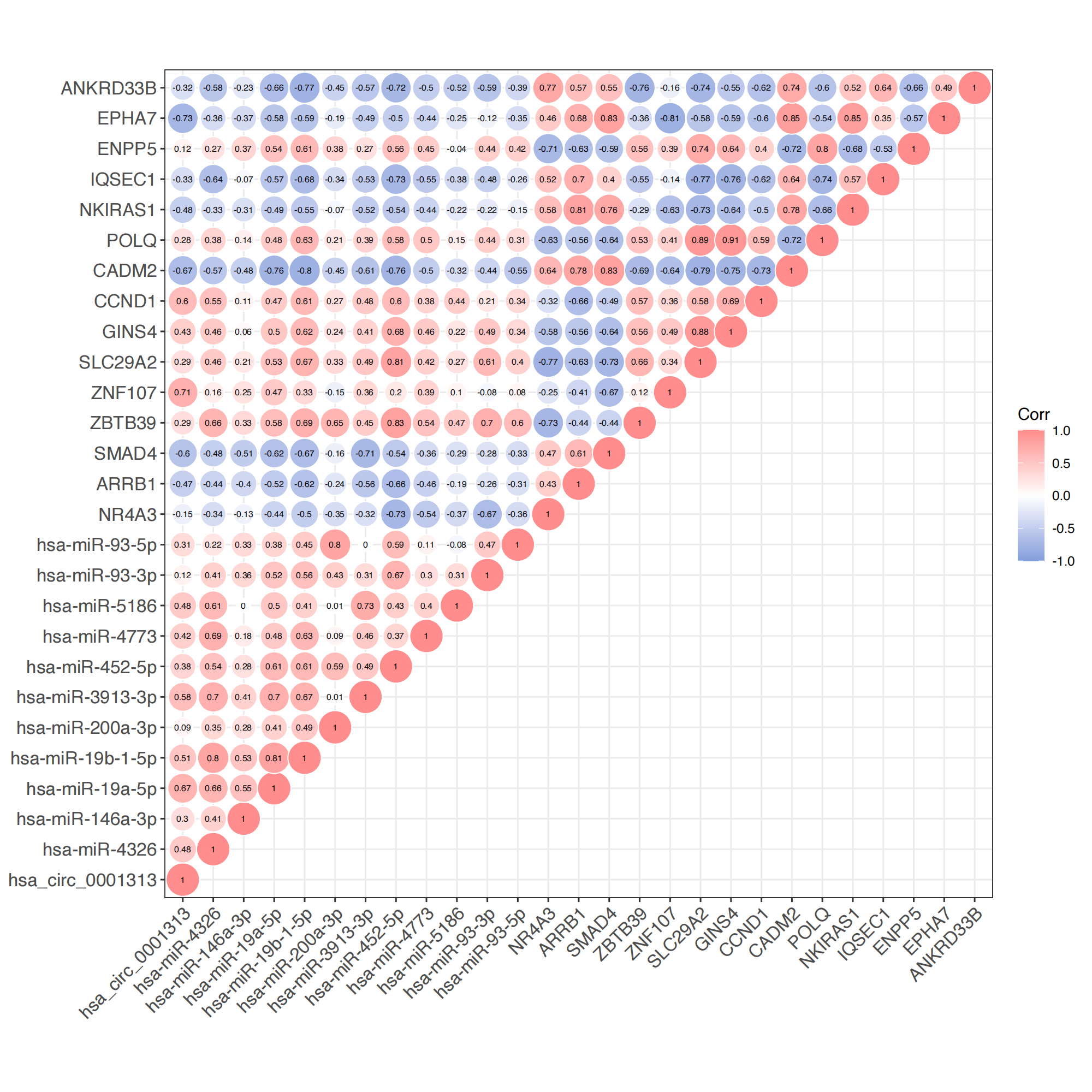


Fig. S 1 Correlation analysis of *circ-CCDC66* (has_circ_0001313) and nodes of its related miRNA and mRNA network by Pearson correlation analysis.
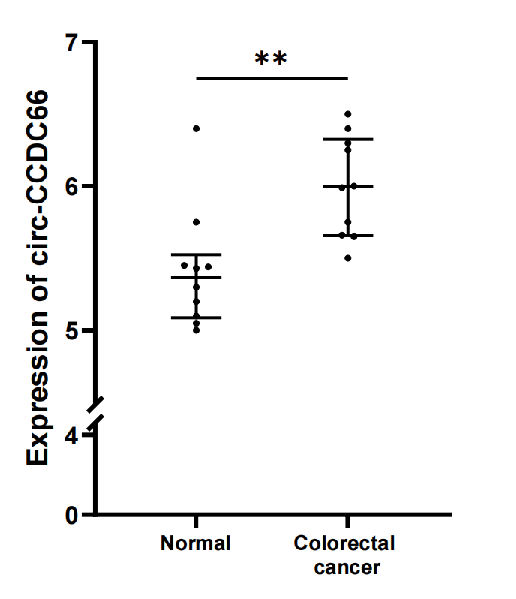


Fig. S 2 Expression of *circ-CCDC66* in colorectal cancer tissues from GSE126094 dataset.Statistical significance was symbolized by ** (p < 0.01).


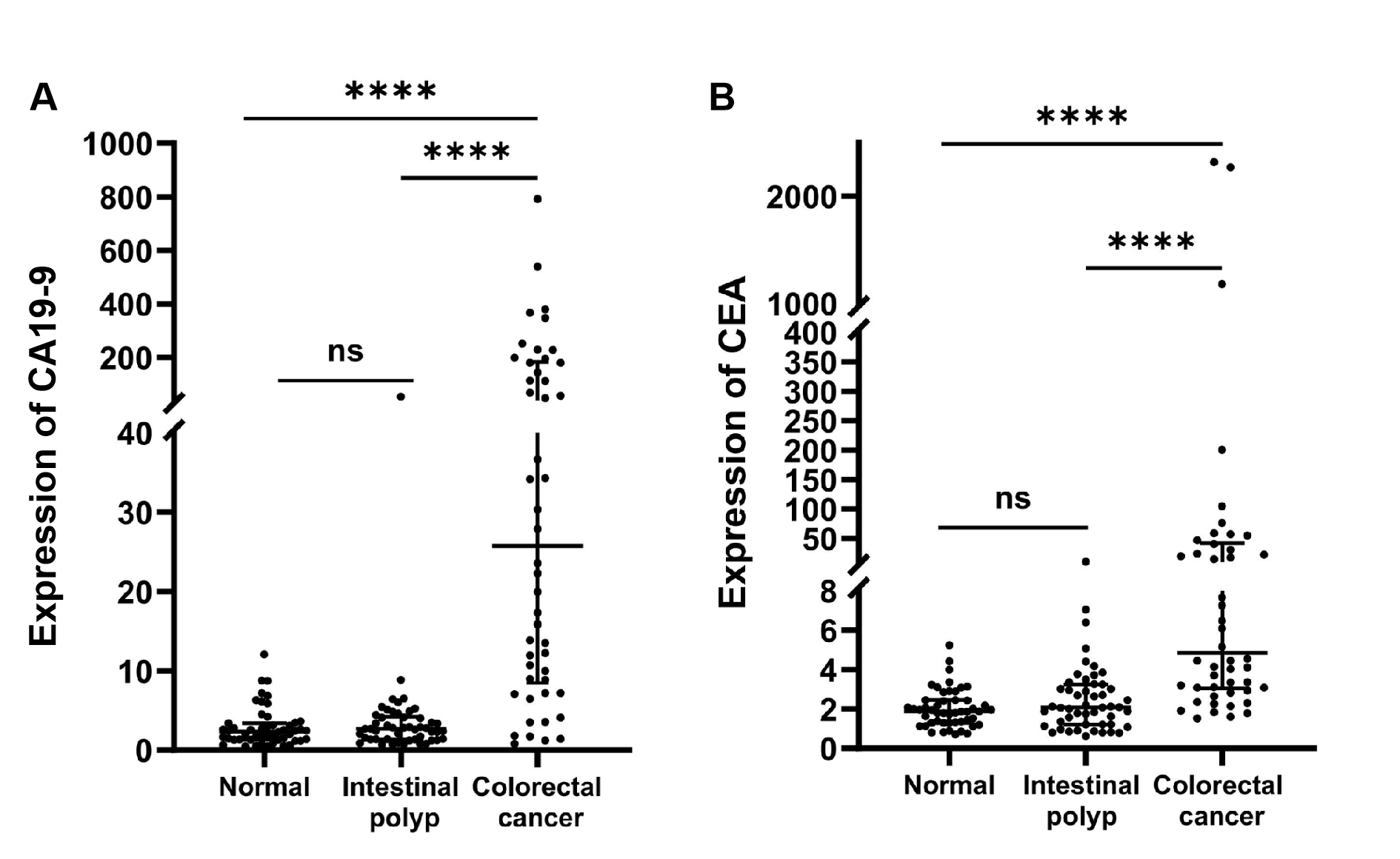


Fig. S 3 Expression levels of (A) CA19-9, and (B) CEA in the three groups of serum samplesby chemiluminescence assay. Statistical significance was symbolized by ns (p > 0.05), * (p <0.05), ** (p < 0.01), *** (p < 0.001), and **** (p < 0.0001)

Table S 5 ROC analysis

| Sample condition | Indicators | AUC | Stand-ard error | 95%  Confidence Interval | | Sensitivity | Specificity |
| --- | --- | --- | --- | --- | --- | --- | --- |
|  |  |  |  | Inferior | Superior |  |  |
| Colorectal cancer (CRC)  &  Normal | circ-CCDC66 | 0.920 | 0.025 | 0.871 | 0.969 | 94% | 72% |
|  | CEA（ng/mL） | 0.887 | 0.032 | 0.825 | 0.950 | 88% | 70% |
|  | CA19-9（U/mL） | 0.907 | 0.033 | 0.843 | 0.971 | 90% | 78% |
|  | circ-CCDC66+CEA +CA19-9 | 0.991 | 0.006 | 0.978 | 1.000 | 98% | 96% |
| Colorectal polyps  & Normal | circ-CCDC66 | 0.855 | 0.036 | 0.784 | 0.926 | 82% | 72% |
|  | CEA（ng/mL） | 0.564 | 0.058 | 0.450 | 0.678 | 40% | 78% |
|  | CA19-9（U/mL） | 0.555 | 0.058 | 0.442 | 0.669 | 52% | 64% |
|  | circ-CCDC66+CEA +CA19-9 | 0.855 | 0.037 | 0.783 | 0.927 | 74% | 78% |
| Colorectal cancer (CRC)  & Colorectal polyps | circ-CCDC66 | 0.683 | 0.054 | 0.578 | 0.789 | 50% | 88% |
|  | CEA（ng/mL） | 0.832 | 0.039 | 0.755 | 0.909 | 62% | 88% |
|  | CA19-9（U/mL） | 0.895 | 0.035 | 0.825 | 0.964 | 84% | 94% |
|  | circ-CCDC66+CEA +CA19-9 | 0.970 | 0.015 | 0.941 | 0.999 | 92% | 94% |

Table S 6 Diagnostic efficacy comparison among different molecular biomarkers in CRC

| Marker | AUC | Sensitivity | Specificity | Ref |
| --- | --- | --- | --- | --- |
| SDC2+ADHFE1+PPP2R5C (mRNA) | 0.93 | 84.8% | 98.0% | [1] |
| miR-874 | 0.818 | 80.8% | 78.6% | [2] |
| miR-874 + CEA | 0.894 | 85.6% | 81.4% |  |
| miR-15b | 0.86 | 81.33% | 91.80% | [3] |
| miR-15b+ miR-21 + miR-31 | 0.913 | 91% | 60% |  |
| miR-16 | 0.79 | 79.05% | 71.55% |  |
| has-miRNA-3937 | 0. 827 | - | - | [4] |
| has-miRNA-3937+CEA +CA199 | 0. 889 | - | - |  |
| miR-99b-5p | 0.733 | - | - | [5] |
| miR-99b-5p  +CEA、CA199、YK1、HSP90 | 0. 84 | - | - |  |
| lncRNA CRNDE-h | 0. 89 | - | - | [6] |
| lncRNA CRNDE-h  +CEA | 0. 91 | - | - |  |
| exosomes LINC02418 | 0. 8978 | 95. 2% | 66. 4% | [7] |
| circ-CCDC66 | 0.920 | 94% | 72% | Our work |
| CEA | 0.887 | 88% | 70% |  |
| CA19-9 | 0.907 | 90% | 78% |  |
| *circ-CCDC66*+CEA+CA19-9 | 0.991 | 98% | 96% |  |

Table S 7 Correlation between the expression level of circ-CCDC66 in CRC plasma and the pathological features.

| **Variable** | **Case Number** | **High expression** | **Low expression** | **Chi square value** (𝜒^2^) | **P-value** |
| --- | --- | --- | --- | --- | --- |
| **Age** |  |  |  | 0.081 | 0.777 |
| ≥64 | 25 | 12 | 13 |  |  |
| ≤64 | 25 | 11 | 14 |  |  |
| **Gender** |  |  |  | 2.92 | 0.087 |
| Man | 28 | 17 | 11 |  |  |
| Female | 22 | 8 | 14 |  |  |
| **Lymphatic Metastasis** |  |  |  | 20.78 | <0.0001 |
| Lymph node metastasis | 28 | 22 | 6 |  |  |
| No Metastasis | 22 | 3 | 19 |  |  |
| **Nerves invasion** |  |  |  | 20.78 | <0.0001 |
| Yes | 22 | 19 | 3 |  |  |
| No | 28 | 6 | 22 |  |  |
| **Tumor size** |  |  |  | 25.93 | <0.0001 |
| >5cm | 24 | 21 | 3 |  |  |
| ≤5cm | 26 | 4 | 22 |  |  |
| **TNM stage** |  |  |  | 36.26 | <0.0001 |
| Ⅰ-Ⅱ | 28 | 4 | 24 |  |  |
| Ⅲ-Ⅳ | 22 | 22 | 0 |  |  |

1. Li, B., et al., *Combined detection of SDC2/ADHFE1/PPP2R5C methylation in stool DNA for colorectal cancer screening.* Journal of Cancer Research and Clinical Oncology, 2023. **149**(12): p. 10241-10253.

2. Zhang, N., et al., *Reduced serum exosomal miR-874 expression predicts poor prognosis in colorectal cancer.* European Review for Medical & Pharmacological Sciences, 2020. **24**(2).

3. Han, L., et al., *Diagnostic value of four serum exosome microRNAs panel for the detection of colorectal cancer.* World Journal of Gastrointestinal Oncology, 2021. **13**(8): p. 970.

4. Qiao, D., et al., *Tumor‐Originated Exosomal hsa‐miR‐3937 as a Minimally Invasive Early Biomarker for Liquid Biopsy of Colorectal Cancer.* Journal of Oncology, 2022. **2022**(1): p. 6990955.

5. Ning, S., et al., *Exosomal miR-99b-5p secreted from mesenchymal stem cells can retard the progression of colorectal cancer by targeting FGFR3.* Stem cell reviews and reports, 2023. **19**(8): p. 2901-2917.

6. Liu, T., et al., *Exosomal long noncoding RNA CRNDE-h as a novel serum-based biomarker for diagnosis and prognosis of colorectal cancer.* Oncotarget, 2016. **7**(51): p. 85551.

7. Zhao, Y., et al., *Long noncoding RNA LINC02418 regulates MELK expression by acting as a ceRNA and may serve as a diagnostic marker for colorectal cancer.* Cell death & disease, 2019. **10**(8): p. 568.
